# Genome-Wide Analysis of Cytochrome P450s of *Alternaria* Species: Evolutionary Origin, Family Expansion and Putative Functions

**DOI:** 10.3390/jof8040324

**Published:** 2022-03-22

**Authors:** Wadzani Palnam Dauda, Daji Morumda, Peter Abraham, Charles Oluwaseun Adetunji, Shakira Ghazanfar, Elkanah Glen, Shittu Emmanuel Abraham, Grace Wabba Peter, Israel Ogwuche Ogra, Ulasi Joseph Ifeanyi, Hannatu Musa, Mawuli Kwamla Azameti, Bilal Ahamad Paray, Aneela Gulnaz

**Affiliations:** 1 Crop Science Unit, Department of Agronomy, Federal University Gashua, Gashua P.M.B. 1005, Yobe State, Nigeria; 2 Department of Microbiology, Federal University Wukari, Wukari P.M.B. 1020, Taraba State, Nigeria; morumda@fuwukari.edu.ng; 3 Department of Horticulture, Federal College of Horticulture, Dadin Kowa P.M.B. 108, Gombe State, Nigeria; peterabraham06@yahoo.com; 4 Applied Microbiology, Biotechnology and Nanotechnology Laboratory, Department of Microbiology, Edo University Iyamho, Auchi P.M.B. 04, Edo State, Nigeria; adetunjicharles@gmail.com; 5 National Agricultural Research Centre, National Institute of Genomics and Agriculture Biotechnology (NIGAB), Park Road, Islamabad 45500, Pakistan; shakira_akmal@yahoo.com; 6 Department of Biochemistry, Federal University Lokoja, Lokoja P.M.B. 1154, Kogi State, Nigeria; elkanahglen@gmail.com; 7 Department of Agronomy, Bayero University Kano, Kano P.M.B. 3011, Kano State, Nigeria; shittu212@gmail.com; 8 Department of Biochemistry, Ahmadu Bello University, Zaria 800001, Kaduna State, Nigeria; petergracewabba@gmail.com (G.W.P.); ograisrael@gmail.com (I.O.O.); 9 Department of Crop Science, University of Uyo, Uyo P.M.B. 1071, Akwa Ibom State, Nigeria; joeulasi@gmail.com; 10 Department of Botany, Ahmadu Bello University, Zaria 800001, Kaduna State, Nigeria; hannatumusa23@gmail.com; 11 Division of Molecular Biology and Biotechnology, Indian Agricultural Research Institute, New Delhi 110012, India; mawuli21@gmail.com; 12 Department of Zoology, College of Science, King Saud University, P.O. Box 2455, Riyadh 11451, Saudi Arabia; bparay@ksu.edu.sa; 13 College of Pharmacy, Woosuk University, Wanju-gun 55338, Korea; draneela@woosuk.ac.kr

**Keywords:** genome-wide, cytochrome P450, *Alternaria* species

## Abstract

Cytochrome P450s are a group of monooxygenase enzymes involved in primary, secondary and xenobiotic metabolisms. They have a wide application in the agriculture sector where they could serve as a target for herbicides or fungicides, while they could function in the pharmaceutical industry as drugs or drugs structures or for bioconversions. *Alternaria* species are among the most commonly encountered fungal genera, with most of them living as saprophytes in different habitats, while others are parasites of plants and animals. This study was conducted to elucidate the diversity and abundance, evolutionary relationships and cellular localization of 372 cytochrome P450 in 13 *Alternaria* species. The 372 CYP proteins were phylogenetically clustered into ten clades. Forty (40) clans and seventy-one (71) cyp families were identified, of which eleven (11) families were found to appear in one species each. The majority of the CYP proteins were located in the endomembrane system. Polyketide synthase (PKS) gene cluster was the predominant secondary metabolic-related gene cluster in all the *Alternaria species* studied, except in *A. porriof*, where non-ribosomal peptide synthetase genes were dominant. This study reveals the expansion of cyps in these fungal genera, evident in the family and clan expansions, which is usually associated with the evolution of fungal characteristics, especially their lifestyle either as parasites or saprophytes, with the ability to metabolize a wide spectrum of substrates. This study can be used to understand the biology, physiology and toxigenic potentials of P450 in these fungal genera.

## 1. Introduction

*Alternaria* species are ubiquitous fungi with different life cycles consisting of saprophytic, endophytic and parasitic modes of living [1]. This genus of fungi is characterized by the formation of large conidia that are usually dark, multicellular, with both transverse and longitudinal septa. *Alternaria* genus is characterized to be in the Family: Pleosporaceae, Order: Pleosporales, Class: Dothediomycetes, Subdivision: Pezizomycotina, Division: Deuteromycotina and Phylum: Ascomycota [2,3]. This genus has been reviewed to consist of about 350 species broadly divided into those with large spores and those with small spores, and which, collectively, have been further subdivided into several sections based on morphological and molecular phylogenetic characteristics [4]. Saprophytic *Alternaria* species play an important ecological role where they collaborate with other microbes to decompose and mineralize plant residues, thereby aiding the bio-geochemical recycling of nutrients. Many *Alternaria* species have been characterized as endophytes residing in healthy plants tissues and producing many bioactive compounds that can stimulate the growth of the host plant, suppress pathogens, improve resistance to environmental stress and aid the assimilation of nitrogen [5,6]. Other members of this genus are important plant pathogens with a broad host range reported to cause diseases and many post-harvest diseases in different crops in about 400 plant species causing significant economic loss to important crops, such as tomatoes, potatoes, apples, etc. [1,7]. Some group members can affect weeds that could be processed and applied as mycoherbicides, while others cause upper respiratory tract infection and asthma in humans [1]. Recent findings have identified *Alternaria* sp. as one of the few fungi capable of degrading untreated extra-heavy crude oil, demonstrating its potential suitability for use in bioremediation [8]. Over 300 secondary metabolites have been described to be produced by the genus *Alternaria*. These belong to different categories of naturally occurring compounds, such as nitrogen-containing compounds, steroids, terpenoids, pyranones (pyrones), quinones and phenolics. They have different biologic activities, such as phytotoxic, cytotoxic and antimicrobial properties, serving as base structures for pesticides and drugs. The biological activities of active compounds have been validated by numerous pharmacologists, plant pathologists and chemists [4,9,10].

Cytochrome P450 are hemoproteins containing monooxygenases that catalyze the transformation of a wide array of endogenous and exogenous substances. They have a wide range of functional properties, such as catalyzing the regio-, chemo- and stereospecific oxidation of a wide array of substrates, indicating their importance as major players in primary and secondary metabolism and xenobiotic degradation [11,12]. Many secondary metabolites that are important to medical, agricultural and industrial processes are biosynthesized with the use of CYPs, of which fungi are producers of a wide spectrum of secondary metabolites making use of CYPs to serve as biocatalyst, drug and agrochemicals targets and for bioremediation of heavily contaminated environment [13,14]. The physiological traits of fungi have been associated with CYPs, such as the pathogenicity of fungi, and it has been reported that the pathogenicity of fungi is a consequence of the expansion and functional diversification of fungal CYPs. CYPs play a housekeeping role in fungi, especially CYP51, which is used in the biosynthesis of sterol, which is a popular antifungal target in the control of human and plant diseases caused by fungi, and they also impact the ecological roles of fungi serving as saprotrophs or decomposers [14]. Therefore, there is a need for an extensive investigation to study the different aspects of CYPs function, regulation and biotechnological applications due to their wide functional and biological roles [13]. Many studies have attempted to analyze the CYPome of many fungi, such as that of *Phanerochete chrysosporium* [15], *Mycosphaerellagraminicola* [16], *Grosmannia clavigera* [17], *Trichoderma* spp., [12] and *Fusarium* spp., [18]. However, this information is scarce in *Alternaria* species and is based on individual species, especially *Alternaria alternata* [19,20,21]. Elucidating the comparative evolutionary process of cytochrome P450 proteins in different *Alternaria* species can further enhance their biotechnological exploration. Therefore, this study intends to perform robust profiling of the CYP in 13 species of *Alternaria* due to the ecological, agricultural, industrial and medical applications, and implications of this important fungal genus.

## 2. Methodology

### 2.1. Sequence Data Retrieval

A total of 1760 cytochrome P450 protein sequences (Supplementary File S1) of 13 species of fungi belonging to the genus *Alternaria* were retrieved from Joint Genome Institute (JGI) fungal genome database MycoCosm (http://genome.jgi-psf.org/programs/fungi/index.jsf (accessed on 18 May 2021)), which are *Alternaria fragaria BMP 3062, A. capsici BMP0180, A. mali BMP3064, A. citriarbusti BMP2343, A. solani BMP0185, A. brassicicola, A. dauci BMP0167, A. tangelonis BMP2327, A. rosae MPI-PUGE-AT-0040 v1.0, A. gaisen BMP2338, A. macrospora BMP1949, A. porri BMP0178* and *A. alternata SRC1lrK2f v1.0*.

### 2.2. Sequence Validation

A two-step procedure was performed for the sequence validation using the procedure established by [19]. Firstly, the retrieved protein sequences of each *Alternaria* spp. were retrieved. Sequences without Cytochrome P450 annotations (as described in the JGI database) were manually removed. Secondly, the conserved domains (CD) of the resultant sequences were further validated in the NCBI batch CD database with the cut-off of positive hits set at E-value 10^−5^ [15]. A total of 372 cyp protein sequences (Supplementary Files S2 and S3) from the 13 *Alternaria* spp. were validated and used for further analyses in the present study.

### 2.3. Annotation of CYPs

The selected fungal cytochrome P450 protein sequence was subjected to blasting on the Fungal Cytochrome P450 database (FCPD) to identify the CYPs families (http://p450.riceblast.snu.ac.kr/blast.php (accessed on 26 June 2021)) on blast program BLOSUM62 matrix with a limited expected value of 1e-5. The predicted sequences were assigned to CYP families and clans to which they have the highest homology (40% and above) from the fungal Cytochrome P450 database (http://p450.riceblast.snu.ac.kr (accessed on 26 June 2021)) against all named fungal CYPs as followed by the International P450 Nomenclature Committee [13].

### 2.4. Construction of Heatmap

An interactive expression heatmap was constructed to show the distribution of the identified Cyp families in the thirteen *Alternaria* species. The data were uploaded to http://heatmapper.ca/expression/ (accessed on 22 February 2022), and the following parameters were used for the heatmap plot: clustering method—average linkage, distance measurement method—Euclidean, scale type row. Clustering was also applied to row while custom color scheme was used.

### 2.5. Phylogenetic Reconstruction of CYPs

Using MEGA X software, the selected fungal cytochrome P450 proteins were subjected to sequence alignment using ClustalW for pairwise and multiple sequence alignment with gaps [22]. The maximum likelihood method and JTT matrix-based model [23] were used to infer the evolutionary history using the neighbor join and BioNJ algorithms to a matrix of pairwise distances estimated using the JTT model, and the topology of the tree was evaluated by bootstrap analysis with one thousand re-sampling replicates. The tree was drawn to scale, with branch lengths measured in the number of substitutions per site. The evolutionary analyses were conducted in MEGA X [22], involving 372 protein sequences following the description of [24].

### 2.6. Identification of Cytochrome P450s Associated with Secondary Metabolism-Related Gene Clusters

This was performed using the automated pipeline on the respective genome pages for all the *Alternaria* species, and the secondary metabolic-related gene clusters included NRPS, PKS, PKS/NRPS, NRPS-like and terpene cyclase clusters.

### 2.7. Subcellular Localization Analysis

BUSCA integrative webserver (https://busca.biocomp.unibo.it (accessed on 26 July 2021)) was used for determining the subcellular localization of the 372 proteins to gain more understanding of the functional mechanism of the Cytochrome P450 proteins [25].

## 3. Results

### 3.1. CYP Proteins in Alternaria

The result obtained shows the presence of 372 cytochrome P450 proteins in the 13 *Alternaria* species in Table 1. It was discovered that *A. macrospora* had the highest number of CYP protein entries (42). This is followed by *A. dauci* and *A. solani,* with 33 and 32 CYP protein entries, respectively. The least number was observed in *A. fragaria,* which had 23, and *A. porri*, with 24 CYP proteins. It was also discovered that a total of 34 cytochrome P450 protein entries had no family matches in the fungal cytochrome P450 database from all the 13 *Alternaria* species, with the majority of this category in *A. brassicicola* (11). In contrast, all the other species had either one, two or three entries with no family match.

### 3.2. Family and Clan Classification

**Figure 1:**
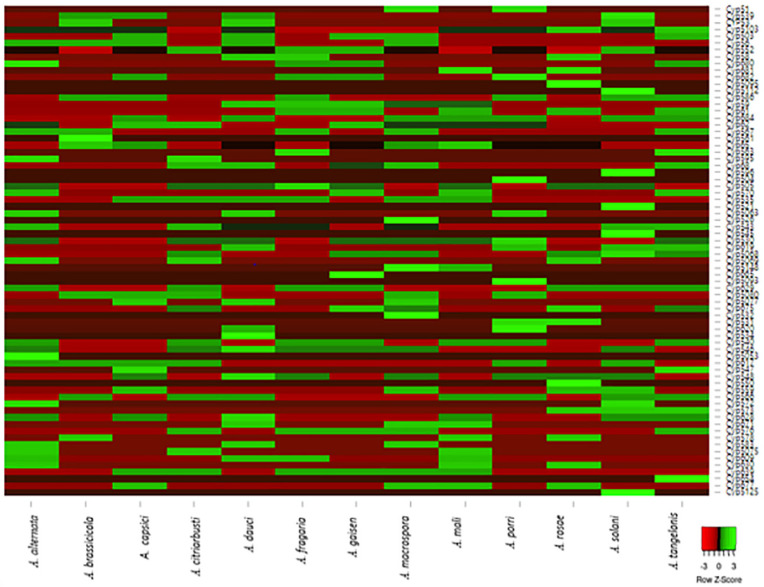
Heatmap showing the distribution of Cyp families (green) or absence (red) across thirteen (13) *Alternaria species.* The data used in generating this heat map are presented in Supplementary Data File S5.

Heatmap showing the distribution of Cyp families (green) or absence (red) across thirteen (13) Alternaria species (Figure 1). The data used in generating this heat map are presented in Supplementary Data File S4. A total of 71 Cyp families and 40 CYP clans were identified in the 13 Alternaria species, as shown in Figure 1. A. macrospora had the most diverse Cyp families (30), followed by A. dauci (27), while the species with the least Cyp family diversity was A. brassicicola (12 families). The results in Figure 1 also show that 11 Cyp families were only found in specific *Alternaria* species. For instance, Cyp532, Cyp526 and Cyp532 were only present in *A. macrospora*, Cyp5093 and Cyp5095 in *A. porri*, Cyp545, Cyp5112 and Cyp596 in *A. solani,* while Cyp665, Cyp521 and Cyp61 were only found in *A. gaisen*, *A. rosae* and *A.* *brassicicola*, respectively. However, Cyp552 was found in 10 *Alternaria* species, showing that it is more conserved than the other Cyps. This is closely followed by Cyp5103, which appeared in nine species, while Cyp65, Cyp505 and Cyp530 were found in eight *Alternaria* species. 

### 3.3. Evolutionary Relationship

**Figure 2:**
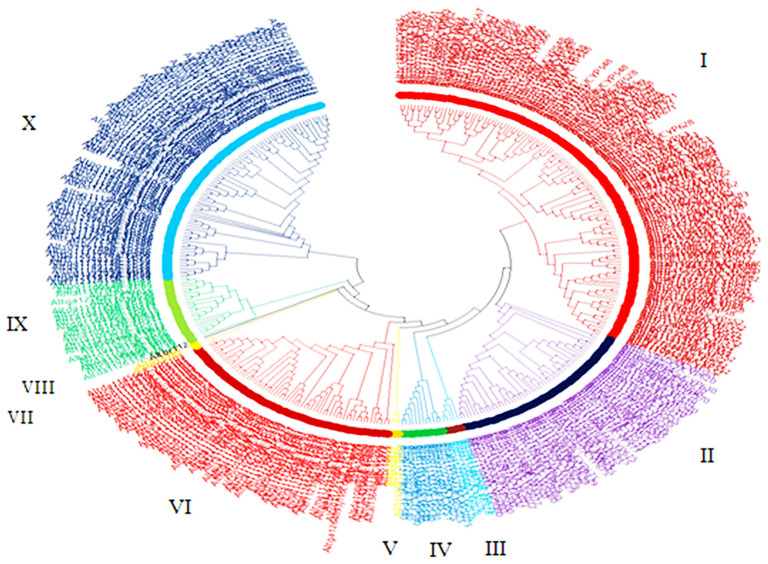
Evolutionary relationship of Cytochrome P450 genes in thirteen *Alternaria* species, which was inferred using MEGA X.

Phylogenetic analysis was carried out using the 372 aligned CYP proteins sequences to demonstrate the evolutionary relationships of the CYPs in the 13 *Alternaria* species, as illustrated in Figure 2. It was discovered that CYPs belonging to the same family, regardless of the *Alternaria* species, were clustered in the same monophyletic clade on the phylogenetic tree, suggesting a strong evolutionary relationship. The different CYPs in these organisms were discovered to be clustered into ten clades, as shown in Table 2. Clade I had the highest branches with 127 CYP proteins entries. Here, 15 CYP proteins with unidentified families were found to be clustered in this clade, and Cyp5095, as the unique Cyp family, was found only in this clade. This is closely followed by clade 10 with 82 phyletic branches having Cyp504, Cyp526, Cyp665 and Cyp5053 as the unique Cyp families found only in this clade. The least number of branching was found in clade VII and clade VIII having two and one branches, respectively; however, all the CYP proteins here had no family match in the FCPD, as shown in Table 2. Individual phylogenetic trees for each of the thirteen (13) species of *Alternaria* are presented in Supplementary File S5.

### 3.4. Subcellular Location

**Figure 3:**
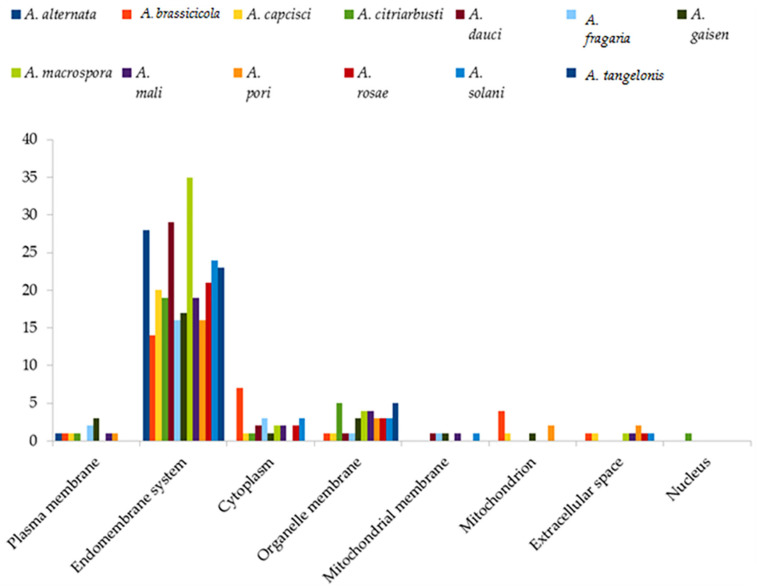
Subcellular localization of cytochrome P450 proteins in thirteen *Alternaria* species.

Subcellular localization of the 372 cytochrome P450 in the 13 *Alternaria* species is presented in Figure 3. Here, it was discovered that most of the Cytochrome P450 proteins were localized in eight subcellular compartments, with the majority located in the endomembrane system (281). The nucleus has the least number of proteins, as only cytochrome P450 proteins of *A. citriarbusti* (1) were located in this organelle.

### 3.5. Distribution of Secondary Metabolite-Related Gene

**Figure 4:**
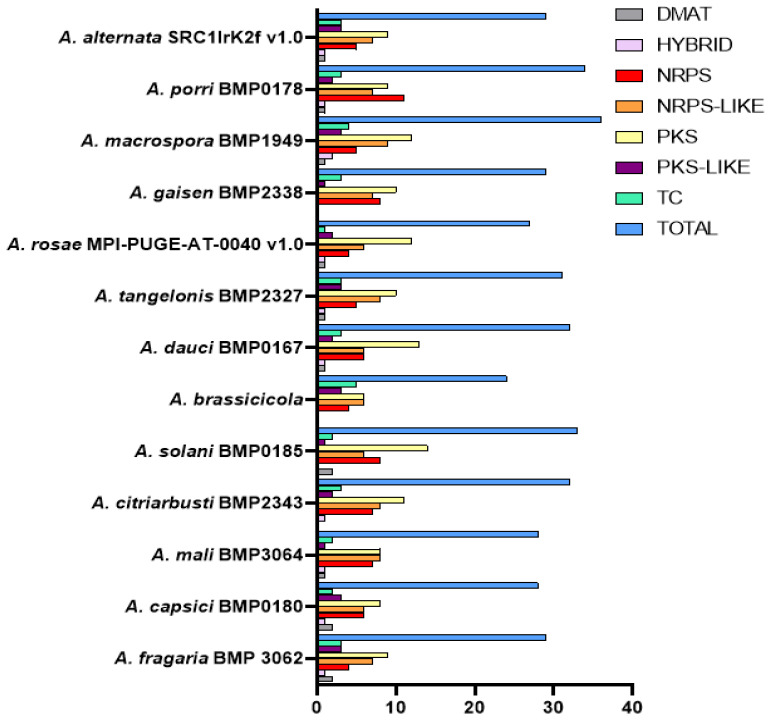
Secondary metabolites-related gene clusters in thirteen *Alternaria* species.

Secondary metabolite gene clusters of the 13 *Alternaria* species are as shown in Figure 4. Here, polyketide synthases (PKSs) had the highest occurrence (131), of which the highest was found in *A. solani* (14). This is followed by non-ribosomal peptide synthetases-like (NRPS-like) (91) with *A. macrospora* having the highest (9), non-ribosomal peptide synthetases (80) with this gene cluster occurring highest in *A. porri* while dimethylallyl diphosphate tryptophan synthases (DMATS), polyketide synthases-like (PKS-like) and terpene cyclases (T.C.) had 13, 27 and 34 occurrences. In contrast, hybrid had the least occurrence (11).

## 4. Discussion

*Alternaria* species are among the most commonly encountered fungi with the greatest global impact on humans and human activities. Many live as saprobes in the various habitats where they are involved in the degradation of a wide diversity of substances, such as leather, marine organisms, plants, wood pulp, sewage, paper, textiles, building supplies, stone monuments, optical instruments, cosmetics, computer disks and jet fuel [26]. CYPs in the *Alternaria* species are distributed into 71 families and 40 clans, which could be due to gene duplication events through evolution, enabling these organisms to survive and live in a wide range of habitats [27,28]. Ref. [14] reported that Cytochrome P450s are important proteins playing diverse biological roles in fungi’s survival and physiological processes, as many have been identified to play a housekeeping role in fungi. It is for these reasons that other members of this genus are plant parasites where they serve as post-harvest pathogens destroying a large amount of agricultural output [1,7], while many others are known to cause human infections, particularly in immuno-compromised patients, causing dermatomycosis, respiratory tract infection, etc., while their spores have been identified as one of the most common and potent sources of both indoor and outdoor allergen [29].

The present study’s findings revealed that some Cyp families were unique to some *Alternaria* species (*A. macrospora, A. porri, A. solani A. gaisen, A. rosae* and *A.brassicicola*). Rampersad (2020) opined that distinct cyps in various fungus species might significantly affect the host specificity of each fungus species to a given plant or animal. *Alternaria* species are well known producers of several host-specific toxins, such as AM-toxin, cercosporin, ABR-toxin, AC-toxin, dothistromin, AB-toxin, Ak-toxin, versicolorin B, Maculosin toxin, AF-toxins, AAL-toxin, AT-toxin, ACT-toxin and AL-toxin, ACR(L)-toxin, HC-toxin, Destuxin A, B, AS-toxin I and AP-toxin [19,20,30,31]), which directly influence their virulence and pathogenicity [21]. Cyp genes were found in all *Alternaria* species investigated. It was believed that the Cyp gene is conserved and plays an important function in *Alternaria*. However, the overall amount found in the examined fungus varies. The findings of our study revealed that five cyp families (Cyp552, Cyp5103, Cyp505, Cyp530 and Cyp65) were predicted to be conserved, as they were shown to be common across the majority of the queried *Alternaria* species, which suggests their significant role in this fungal genus. The four cyp families (Cyp552, Cyp505, Cyp530 and Cyp65) were previously reported in *Aspergillus nidulans* [32], *Grosmannia clavigera* [17], *Mycosphaerella graminicola* [16] and *Trichoderma harzianum* [12], while Cyp505 was reported in *Phanerochete chrysosporium* [15]. However, the Cyp5103 family predicted in 8 out of the 13 *Alternaria* spp. was not reported in any of the aforementioned fungal genera, which implies that this cyp family could serve as a vital target to be harnessed for their management or biosynthesis of important metabolites in these fungi. Cyp51 and Cyp61, reported to be common in both plant and animal species, were predicted in only 2 of the 13 queried *Alternaria* species. The spread and clustering of Cyp families into 10 phyletic clades across the 13 selected *Alternaria* species suggest several expansions and narrowing of cyp families along a paralogous evolutionary path, which could favor the development of several fungal traits to ensure the successful adaptation and colonization of their environment, including pathogenicity, as observed by [33,34]. Fungal cyp family expansions and functional diversifications have been linked to the development of fungal pathogenicity [29]. Despite some parallels in CYPome distribution amongst the *Alternaria* species, the family diversity of cyp genes varies significantly between the species. It is represented in their family number and in their family kind. We believe that the diversity in the cyp genes among the *Alternaria* species is connected to the potential need for novel physiological activities. The study revealed the different putative functions engaged by each phyletic clade of the 13 *Alternaria* spp. The beneficial roles played by p450s genes in various cell functions, including neutralization of host defense, metabolism of xenobiotics and primary and secondary metabolism, have been well documented [12]. The phylogeny of all annotated cyps produced revealed several cyp branches in the phylogenetic tree, demonstrating their significantly evolved divergence.

The findings of this study also established the localization of cyps genes in eight different cellular components (plasma membrane, cytoplasm, endomembrane system, organelle membrane, mitochondrion, mitochondrial membrane, extracellular space and nucleus) of *Alternaria* spp., which suggests their varying important roles in the species. Fungal species are known to possess the class II enzyme group, characterized to be involved in diverse cellular functions, such as biosynthesis of secondary metabolites (mycotoxins), lipid metabolism, sterols of membranes, detoxification of xenobiotics and phytoalexins, therefore, justifying the multicellular localization of their cyps [35,36].

Additionally, the role of P450 genes in the biosynthesis of secondary metabolites (S.M.), such as mycotoxins in fungal species, has been well documented [36]. Even though these S.M.s have not been shown to have a direct effect on the growth and development of fungi [37], they are significant for the colonization of their environment by serving as growth inhibitors of their competitors and chemical communicating signals [38,39,40]. The pathogenesis of several fungal pathogens is aided by the secondary metabolite they biosynthesized [41]. *Alternaria* species are notable producers of secondary metabolites, of which the majority are powerful mycotoxins known to be involved in cancer development. For this, over 300 known secondary metabolites are known to belong to either steroids, terpenoids, pyranones (pyrones), quinones and phenolics, identified to exhibit different biologic activities, such as phytotoxic, cytotoxic and antimicrobial properties, serving as base structures for pesticides and drugs, and many of these are biosynthesized by CYP proteins [4,14,32,42,43]. This is evident in this study’s discovery of the preponderance of polyketide synthase (PKS) secondary metabolic-related gene cluster in all the *Alternaria* species studied, which is implicated in building the structural support of many secondary metabolites as they are multi-domain and multi-functional enzymes [41]. Other structural genes reported to aid in the synthesis of secondary metabolites include P450 monooxygenases, methyltransferases, reductases, dimethylallyl tryptophan synthase (DMATS), polyketide synthase-like (PKS-like), polyketide synthase (PKS), non-ribosomal peptide synthase (NRPS), non-ribosomal peptide synthase-like (NRPS-like) and terpene cyclases (T.C.) [40,44,45,46] established the active role of non-ribosomal peptide synthase genes (NPS6, AbNPS2) to be involved in the biosynthesis of secondary metabolites, which directly promotes the integrity of the cell wall, viability of conidia, virulence of old spore and pathogenicity in *A. brassicicola.* Following the recent identification of 12 cercosporin toxin biosynthesis (CTB) genes from the whole-genome sequencing of *A*. *alternata* (Y784-BC03), a pathway for the biosynthesis of cercosporin was postulated, which was regulated by non-reducing polyketide synthase [21]. These studies indicate the significant importance of these secondary metabolites in fungi. 

## 5. Conclusions

Our analysis has revealed the various Cytochrome P450 clans and families in the 13 *Alternaria* species and their distribution into the different phylogenetic groups, including their putative functions in metabolism. Here, 372 CYP proteins were identified to belong to 71 Cyp families, revealing the diverse biological, agricultural and biotechnological potential of these fungi. These fungi are a group of commonly encountered genera living in different habitats and utilizing a diverse substrate spectrum. The phylogenetic clustering of these proteins into ten clades reveals their close relationships and further demonstrates the influence of gene expansion and duplication during the evolutionary process. The majority of these proteins were identified to be located in the endo-membrane system, revealing intense participation of these proteins in active synthesis, packaging and transportation of substances in the cell. Their potential can be harnessed in bio-conversion and transformation of different compounds. The occurrence of secondary metabolite gene clusters is further evidence revealing the involvement of these genera in the synthesis of diverse secondary metabolites with agricultural, pharmaceutical, medical and industrial applications. This study will enable the selection of *Alternaria* species for various agricultural, medical and biotechnological applications, including their use to clean environmental pollution.

## Figures and Tables

**Table 1:** Taxonomic distribution of putative CYPs in thirteen *Alternaria* species.

*Alternaria* Species	Genome Size (Mb)	Number of Predicted Genes	Total Cyp Proteins	Protein with Complete Sequences	Family Type	Clan Type	Families with No FCPD Matches
*A. fragaria* BMP 3062	33,135,386	12,272	125	23	19	14	1
*A. capsici* BMP0180	31,350,549	11,487	113	25	20	15	3
*A. mali* BMP3064	34,331,800	12,715	132	28	23	19	3
*A. citriarbusti* BMP2343	33,865,016	12,606	131	27	21	16	2
*A. solani* BMP0185	31,129,923	12,258	129	32	24	23	1
*A. brassicicola*	31,974,449	10,688	127	28	12	10	11
*A. dauci* BMP0167	30,427,686	11,981	123	33	27	23	1
*A. tangelonis* BMP2327	33,765,687	12,639	125	28	24	19	1
*A. rosae* MPI-PUGE-AT-0040 v1.0	33,831,682	12,640	152	27	19	14	5
*A. gaisen* BMP2338	33,998,619	13,902	137	26	20	15	2
*A. macrospora* BMP1949	31,350,355	11,961	130	42	30	22	2
*A. porri* BMP0178	29,243,729	12,232	127	24	20	15	1
*A. alternata* SRC1lrK2f v1.0	32,990,834	13,469	209	29	26	22	1
Total			1760	372			34

**Table 2:** Phylogenetic clustering of Cytochrome P450 families and clans among thirteen *Alternaria* spp.

Phylogenetic Clade	Sequence Entry	CYP Families	CYP Clans	Putative Function
I	127	Cyp65, Cyp561, Cyp563, Cyp62, Cyp567, Cyp566, Cyp548, Cyp528, Cyp539, Cyp628, Cyp671, Cyp53, Cyp673, Cyp684, Cyp583, Cyp578, Cyp680, Cyp643, Cyp5095, Cyp677, Cyp682, Cyp681, Cyp552	CYP65, CYP61, CYP62, CYP566, CYP548, CYP528, CYP52, CTP574, CYP53, CYP673, CYP583, CYP578, CYP58, CYP643, CYP677	Xenobiotic metabolism, Secondary metabolism, Primary metabolism
II	53	Cyp561, Cyp620, Cyp630, Cyp570, Cyp527, Cyp535, Cyp573, Cyp675, Cyp629, Cyp5080, Cyp531, Cyp5077, Cyp532	CYP65, CYP533, CYP630, CYP507, CYP572, CYP531	Xenobiotic metabolism, secondary metabolism Primary metabolism
III	5	Cyp576	CYP576	
IV	12	Cyp5075, Cyp51, Cyp540	CYP589, CYP51, CYP540	Primary metabolism
V	3	Cyp539	CYP52	Xenobiotic metabolism
VI	63	Cyp609, Cyp5112, Cyp561, Cyp530, Cyp645, Cyp639, Cyp550, Cyp61, Cyp5125, Cyp559, Cyp68, Cyp595, Cyp596, Cyp503, Cyp654, Cyp5103	CYP609, CYP58, CYP530, CYP65, CYP645, CYP639, CYP550, CYP61, CYP559, CYP68, CYP54, CYP653	Secondary metabolism, Xenobiotic metabolism, Primary metabolism
VII	2	No match in FCDP	No match in FCDP	
VIII	1	No match in FCDP	No match in FCDP	
IX	24	Cyp534, Cyp56, Cyp547, Cyp539, Cyp65,	CYP534, CYP56, CYP547, CYP52, CYP65	Xenobiotic metabolism, Primary metabolism, Secondary metabolism
X	82	Cyp59, Cyp586, Cyp526, Cyp505, Cyp504, Cyp665, Cyp530, Cyp546, Cyp5053, Cyp5093, Cyp5068, CYP5063, CYP5069, CYP5148, CYP620, CYP530, CYP628, CYP543	CYP59, CYP526, CYP505, CYP504, CYP52, CYP530, CYP546 CYP5063, CYP533, CYP529	Xenobiotic metabolism, Primary metabolism, Secondary metabolism

## Data Availability

Data is contained within the article or Supplementary Material.

## Supplementary Materials

The following supporting information can be downloaded at: https://www.mdpi.com/article/10.3390/jof8040324/s1, supplementary data file S1: Total Cytochrome P450 Protein gene sequence of 13 Alternaria species retrieved from JGI server); supplementary data file S2: Validated Cytochrome P450 Protein gene sequences of 13 Alternaria species used for further analysis, supplementary data file S3: 372 validated cyp protein sequences of 13 Alternaria spp used for analyses, supplementary data file S4: figures of individual phylogenetic tree of 13 Alternaria species, Supplementary data file S5: Distribution of Cyp families in 13 Alternaria species used in generating heatmap.

## References

[1] Meena M., Gupta S.K., Swapnil P., Zehra A., Dubey M.K., Upadhyay R.S. (2017). Alternaria toxins: Potential virulence factors and genes related to pathogenesis. Front. Microbiol..

[2] Mamgain A., Roychowdhury R., Tah J. (2013). Alternaria pathogenicity and its strategic controls. Res. J. Biol..

[3] Singh V., Shrivastava A., Jadon S., Wahi N., Singh A., Sharma N. (2015). Alternaria diseases of vegetable crops and its management control to reduce the low production. Int. J. Agric. Sci..

[4] Dalinova A.A., Salimova D.R., Berestetskiy A.O. (2020). Fungi of the genera Alternaria as producers of biological active compounds and mycoherbicides. Appl. Biochem. Microbiol..

[5] Patil S.V., Jayamohan N.S., Kumudini B.S. (2016). Strategic assessment of multiple plant growth promotion traits for shortlisting of fluorescent *Pseudomonas* spp. and seed priming against ragi blast disease. Plant Growth Regul..

[6] Elgorban A.M., Bahkali A.H., Al Farraj D.A., Abdel-Wahab M.A. (2019). Natural products of Alternaria sp., an endophytic fungus isolated from *Salvadora persica* from Saudi Arabia. Saudi J. Biol. Sci..

[7] Sajad A.M., Jamaluddin, Abid H.Q. (2017). Fungi associated with the spoilage of post-harvest tomato fruits and their frequency of occurrences in different markets of Jabalpur, Madhya-Pradesh, India. Int. J. Curr. Res. Rev..

[8] Romero-Hernández L., Velez P., Betanzo-Gutiérrez I., Camacho-López M.D., Vázquez-Duhalt R., Riquelme M. (2021). Extra-heavy crude oil degradation by *Alternaria* sp. isolated from deep-sea sediments of the Gulf of Mexico. Appl. Sci..

[9] Lou J., Fu L., Peng Y., Zhou L. (2013). Metabolites from Alternaria fungi and their bioactivities. Molecules.

[10] Zarafi A.B., Dauda W.P. (2019). Exploring the importance of fungi in agricultural biotechnology. Int. J. Agric. Sci. Vet. Med..

[11] Takaoka S., Kurata M., Harimoto Y., Hatta R., Yamamoto M., Akimitsu K., Tsuge T. (2014). Complex regulation of secondary metabolism controlling pathogenicity in the phytopathogenic fungus Alternaria alternata. New Phytol..

[12] Chadha S., Mehetre S.T., Bansal R., Kuo A., Aerts A., Grigoriev I.V., Druzhinina I.S., Mukherjee P.K. (2018). Genome-wide analysis of cytochrome P450s of Trichoderma spp.: Annotation and evolutionary relationships. Fungal Biol. Biotechnol..

[13] Kelly S.L., Kelly D.E. (2013). Microbial cytochromes P450: Biodiversity and biotechnology. Where do cytochromes P450 come from, what do they do and what can they do for us?. Philos. Trans. R. Soc. B Biol. Sci..

[14] Chen W., Lee M.K., Jefcoate C., Kim S.C., Chen F., Yu J.H. (2014). Fungal cytochrome p450 monooxygenases: Their distribution, structure, functions, family expansion, and evolutionary origin. Genome Biol. Evol..

[15] Syed K., Yadav J.S. (2012). P450 monooxygenases (P450ome) of the model white rot fungus *Phanerochaete chrysosporium*. Crit. Rev. Microbiol..

[16] Newsome A.W., Nelson D., Corran A., Kelly S.L., Kelly D.E. (2013). The cytochrome P 450 complement (CYP ome) of *Mycosphaerella graminicola*. Biotechnol. Appl. Biochem..

[17] Lah L., Haridas S., Bohlmann J., Breuil C. (2013). The cytochromes P450 of Grosmannia clavigera: Genome organization, phylogeny, and expression in response to pine host chemicals. Fungal Genet. Biol..

[18] Dauda W.P., Glen E., Abraham P., Adetunji C.O., Morumda D., Abraham S.E., Wabba G.P., Ogwuche I.O., Azameti M.K. (2021). Comparative Phylogenomic Analysis of Cytochrome P450 Monooxygenases from Fusarium Species.

[19] Tsuge T., Harimoto Y., Hanada K., Akagi Y., Kodama M., Akimitsu K., Yamamoto M. (2016). Evolution of pathogenicity controlled by small, dispensable chromosomes in Alternaria alternata pathogens. Physiol. Mol. Plant Pathol..

[20] Meena M., Samal S. (2019). Alternaria host-specific (HSTs) toxins: An overview of chemical characterization, target sites, regulation and their toxic effects. Toxicol. Rep..

[21] Huang K., Tang J., Zou Y., Sun X., Lan J., Wang W., Xu P., Wu X., Ma R., Wang Q. (2021). Whole-genome sequence of Alternaria alternata, the causal agent of black spot of kiwifruit. Front. Microbiol..

[22] Kumar S., Stecher G., Li M., Knyaz C., Tamura K. (2018). MEGA X: Molecular evolutionary genetics analysis across computing platforms. Mol. Biol. Evol..

[23] Jones D.T., Taylor W.R., Thornton J.M. (1992). The rapid generation of mutation data matrices from protein sequences. Bioinformatics.

[24] Dauda W.P., Abraham P., Fasogbon I.V., Adetunji C.O., Banwo O.O., Kashina B.D., Alegbejo M.D. (2021). Cassava mosaic virus in Africa: Functional analysis of virus coat proteins based on evolutionary processes and protein structure. Gene Rep..

[25] Savojardo C., Martelli P.L., Fariselli P., Profiti G., Casadio R. (2018). BUSCA: An integrative web server to predict subcellular localization of proteins. Nucleic Acids Res..

[26] Dang H.X., Pryor B., Peever T., Lawrence C.B. (2015). The Alternaria genomes database: A comprehensive resource for fungal genus comprised of saprophytes, plant pathogens, and allergic species. BMC Genom..

[27] Kgosiemang I.K.R., Mashele S.S., Syed K. (2014). Comparative genomics and evolutionary analysis of cytochrome P450 monooxygenases in fungal subphylum Saccharomycotina. J. Pure Appl. Microbiol..

[28] Shin J., Kim J.E., Lee Y.W., Son H. (2018). Fungal cytochrome P450s and the P450 complement (CYPome) of *Fusarium graminearum*. Toxins.

[29] Soanes D.M., Alam I., Cornell M., Wong H.M., Hedeler C., Paton N.W., Rattray M., Hubbard S.J., Oliver S.G., Talbot N.J. (2008). Comparative genome analysis of filamentousfungi reveals gene family expansions associated with fungal pathogen-esis. PLoS ONE.

[30] Nishimura S., Kohmoto K. (1983). Host-specific toxins and chemical structures from Alternaria species. Annu. Rev. Phytopathol..

[31] Tsuge T., Harimoto Y., Akimitsu K., Ohtani K., Kodama M., Akagi Y., Egusa M., Yamamoto M., Otani H. (2013). Host-selective toxins produced by the plant pathogenic fungus Alternaria alternata. FEMS Microbiol. Rev..

[32] Kelly D.E., Kraševec N., Mullins J., Nelson D.R. (2009). The CYPome (cytochrome P450 complement) of Aspergillus nidulans. Fungal Genet. Biol..

[33] Syed K., Shale K., Pagadala N.S., Tuszynski J. (2014). Systematic identification and evolutionary analysis of catalytically versatile cytochrome p450 monooxygenase families enriched in model basidiomycete fungi. PLoS ONE.

[34] Hansen C.C., Nelson D.R., Møller B.L., Werck-Reichhart D. (2021). Plant cytochrome P450 plasticity andevolution. Mol. Plant.

[35] Nelson D.R., Koymans L., Kamataki T., Stegeman J.J., Feyereisen R., Waxman D.J., Waterman M.R., Gotoh O., Coon M.J., Estabrook R.W. (1996). P450 superfamily: Update on new sequences, gene mapping, accession numbers and nomenclature. Pharmacogenetics.

[36] Werck-Reichhart D., Feyereisen R. (2000). Cytochromes P450: A success story. Genome Biol..

[37] Brakhage A.A. (2012). Regulation of fungal secondary metabolism. Nat. Rev. Microbiol..

[38] Adetunji C.O., Olaniyan O.T., Anani O.A., Inobeme A., Ukhurebor K.E., Bodunrinde R.E., Adetunji J.B., Singh K.R., Nayak V., Palnam W.D. (2021). Bionanomaterials for green bionanotechnology. Bionanomaterials.

[39] Yim G., Wang H.H., Frs J.D. (2007). Antibiotics as signalling molecules. Philos. Trans. R. Soc. B Biol. Sci..

[40] Brakhage A.A., Schroeckh V. (2011). Fungal secondary metabolites–strategies to activate silent gene clusters. Fungal Genet. Biol..

[41] Saha P., Sarkar A., Sabnam N., Shirke M.D., Mahesh H.B., Nikhil A., Rajamani A., Gowda M., Roy-Barman S. (2021). Comparative analysis of secondary metabolite gene clusters in different strains of Magnaporthe oryzae. FEMS Microbiol. Lett..

[42] Thomma B.P. (2003). Alternaria spp.: From general saprophyte to specific parasite. Mol. Plant Pathol..

[43] Oyekanmi A., Okibe F., Dauda W.P. (2018). Toxic elements levels in water and some vegetable crops grown in farms in Bade Local Government area of Yobe state, Nigeria. Asian J. Phys. Chem. Sci..

[44] Adetunji C.O., Olaniyan O.T., Igere B.E., Ekundayo T.C., Anani O.A., Bodunrinde R.E., Olisaka F.N., Inobeme A., Uwadiae E.O., Obayagbona O.N. (2021). Microbial Degradation of Chlorophenolic Compounds. Recent Advances in Microbial Degradation.

[45] Oide S., Moeder W., Krasnoff S., Gibson D., Haas H., Yoshioka K., Turgeon B.G. (2006). NPS6, encoding a non-ribosomal peptide synthetase involved in siderophore-mediated iron metabolism, is a conserved virulence determinant of plant pathogenic ascomycetes. Plant Cell.

[46] Kim K.H., Cho Y., La Rota M., Cramer R.A., Lawrence C.B. (2007). Functional analysis of the Alternaria brassicicola non-ribosomal peptide synthetase gene AbNPS2 reveals a role in conidial cell wall construction. Mol. Plant Pathol..

