# Supplementary material for: Genome-Wide Analysis of Cytochrome P450s of Alternaria Species: Evolutionary Origin, Family Expansion and Putative Functions
Source: J Fungi (Basel). 2022 Mar 22;8(4):324. doi: 10.3390/jof8040324 (PMC9028179; doi:10.3390/jof8040324)
Supplement: Supplementary file 1 [file jof-08-00324-s001.zip › Supplementary data file S4(figures of individual phylogenetic tree).pdf]

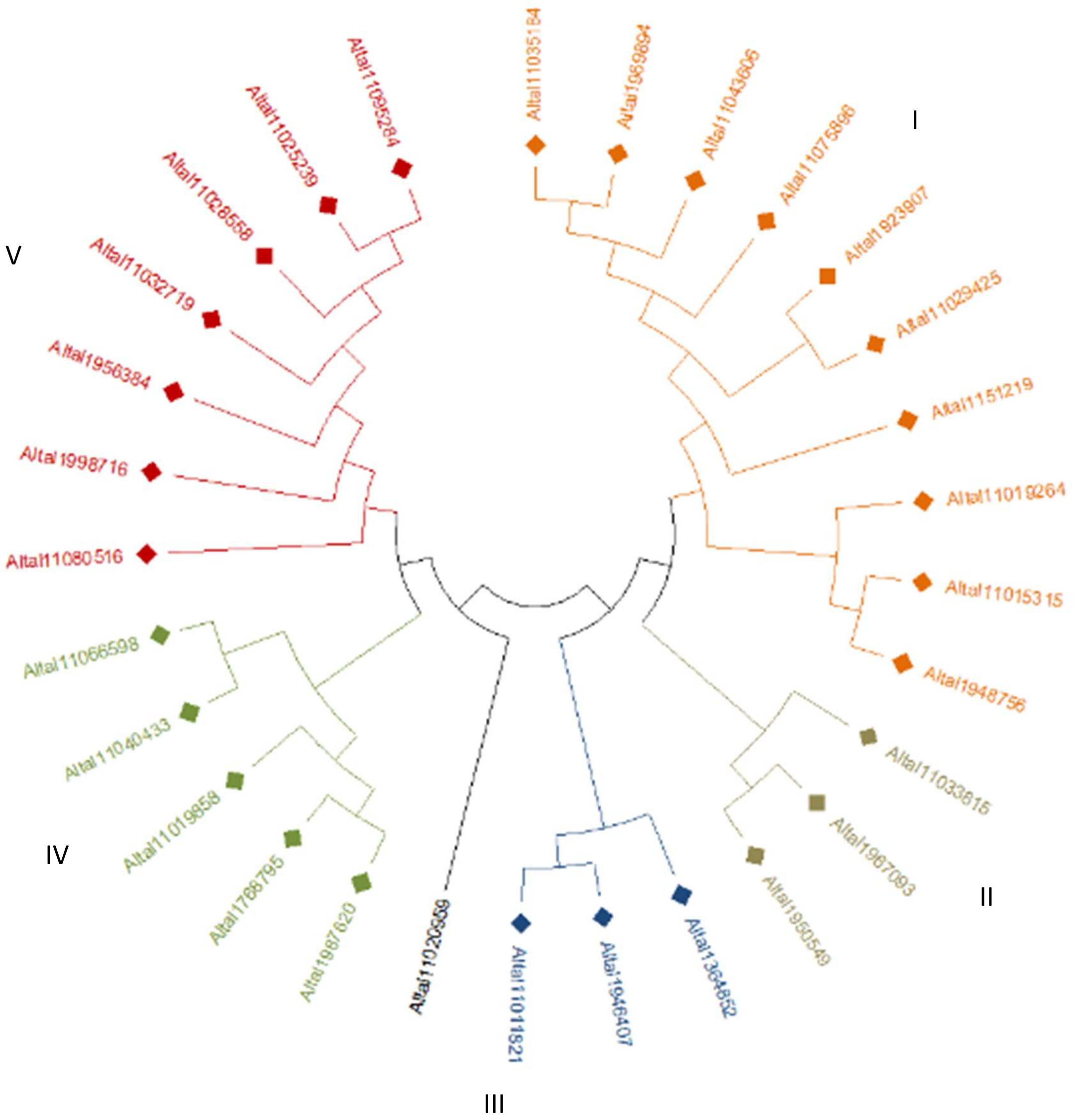

Alternaria alternata

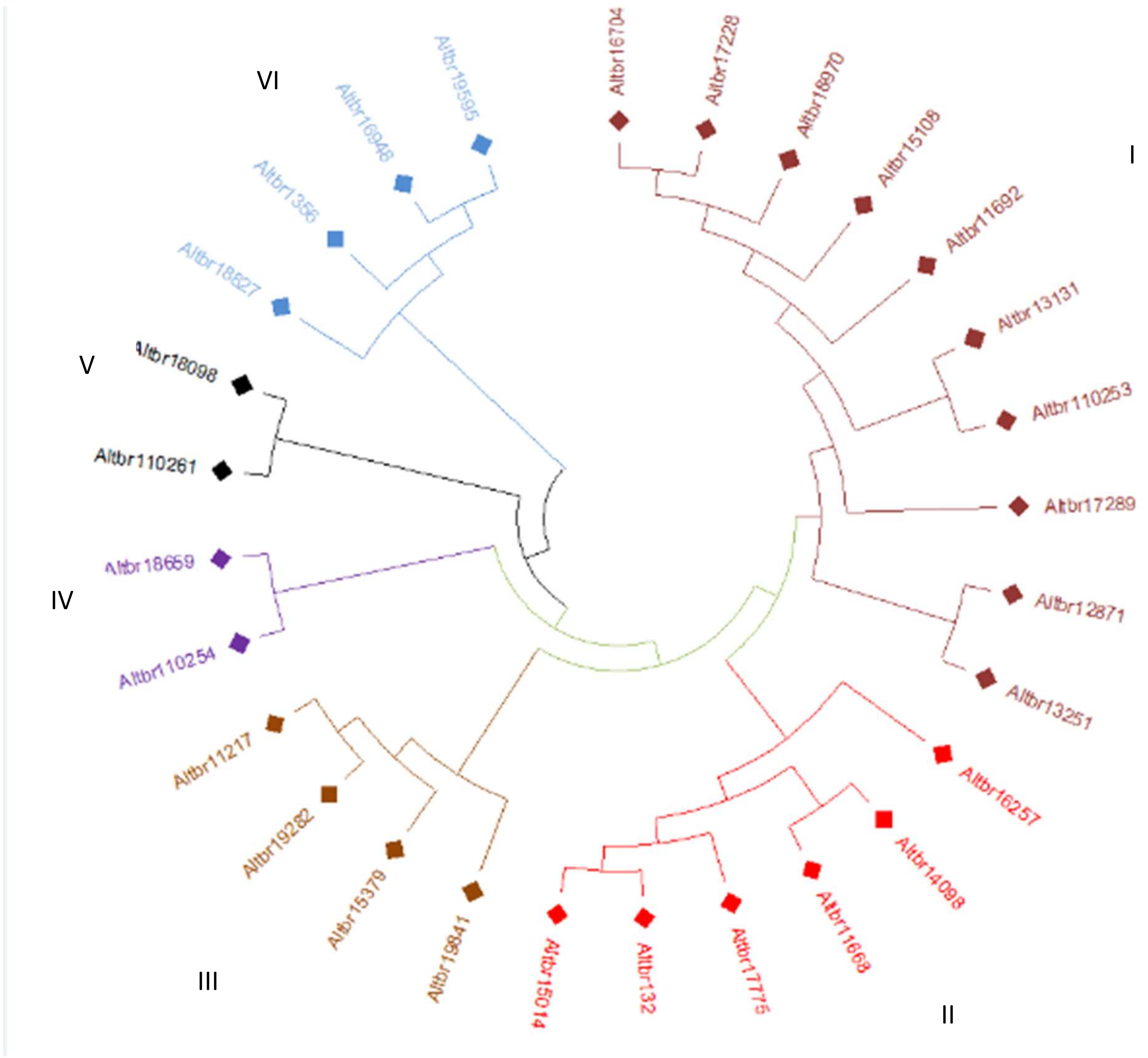

Alternaria brasissicola

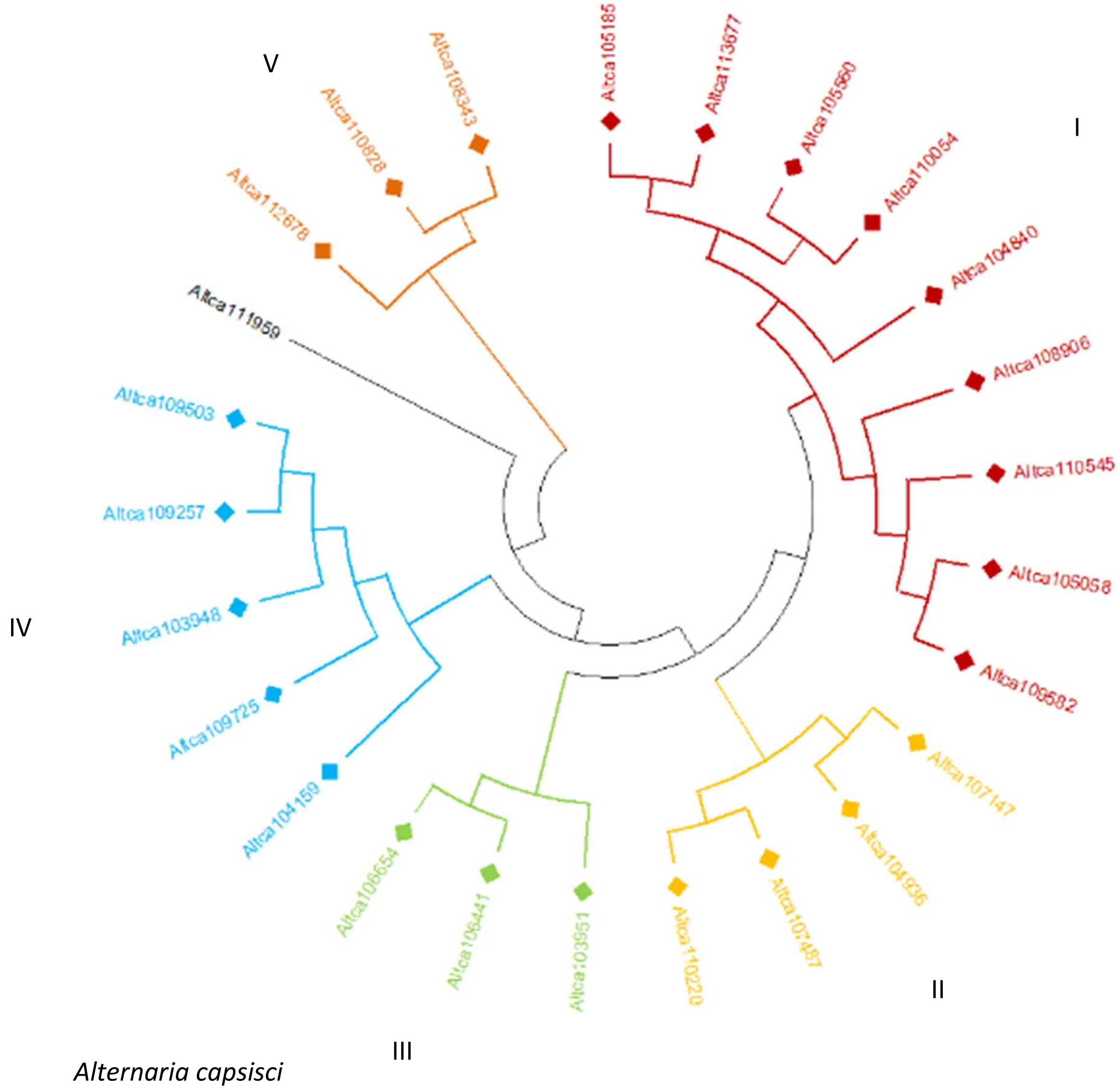

Alternaria capsisci

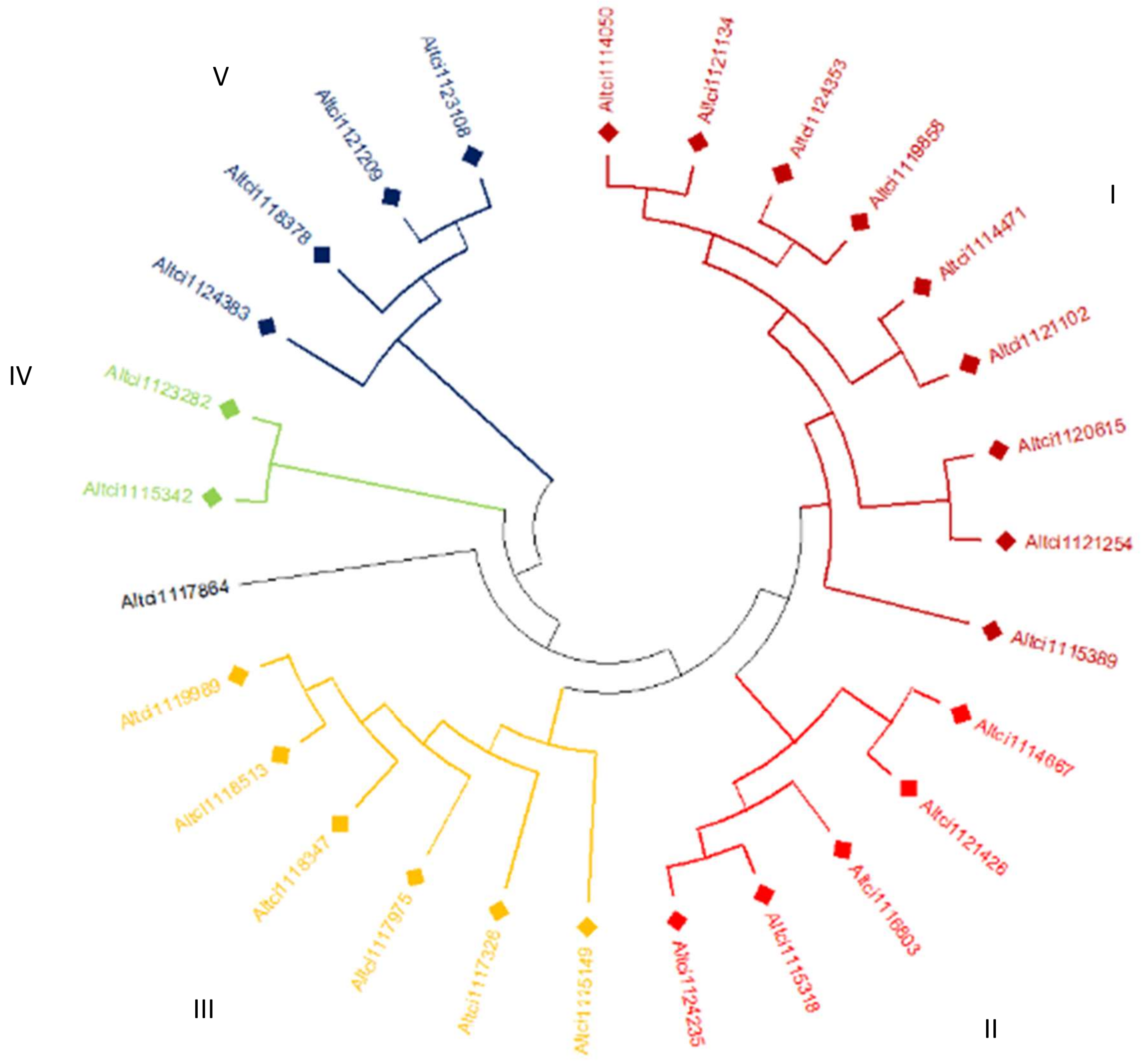

Alternaria citriarbusti

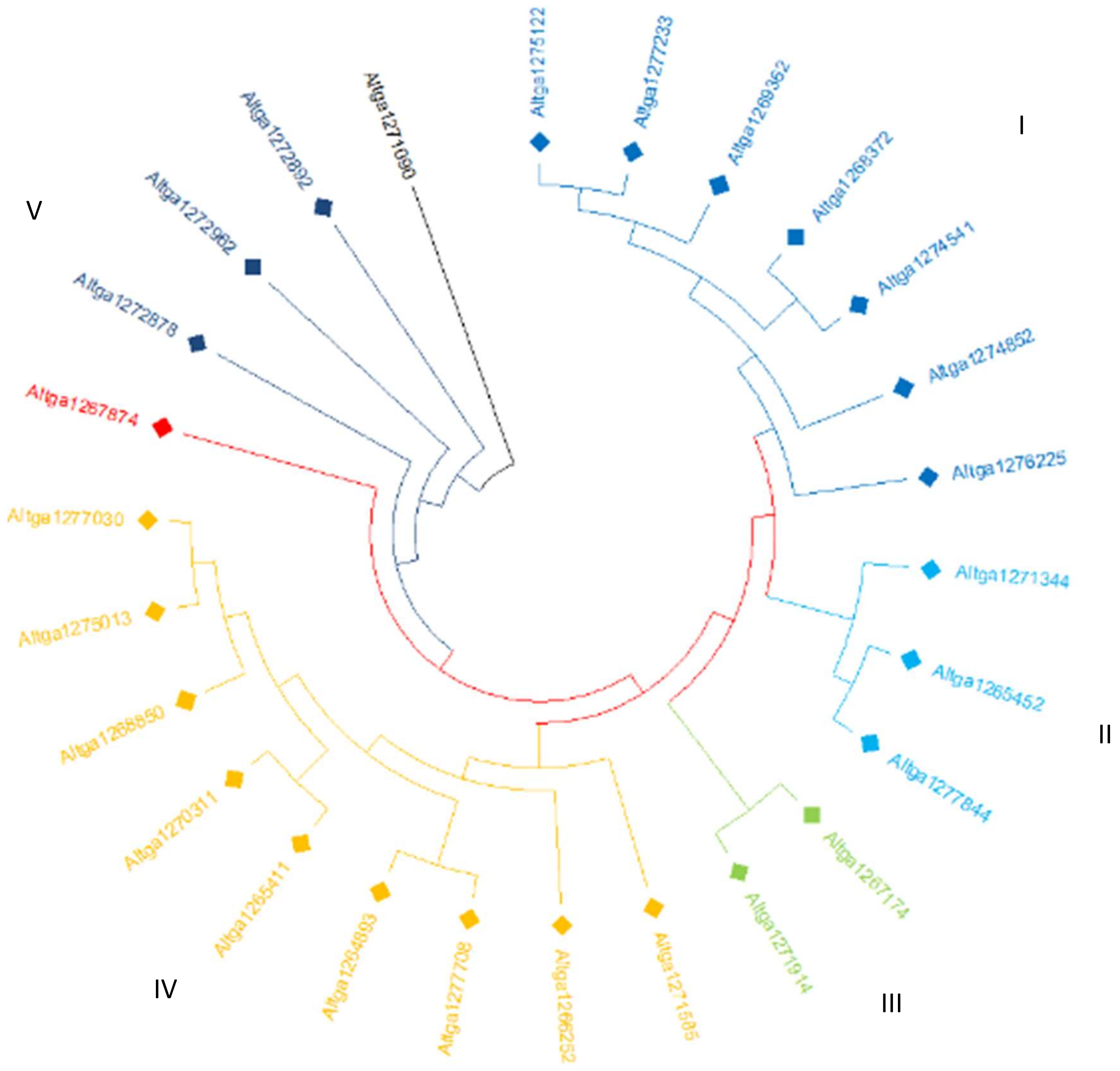

Alternaria gaisen

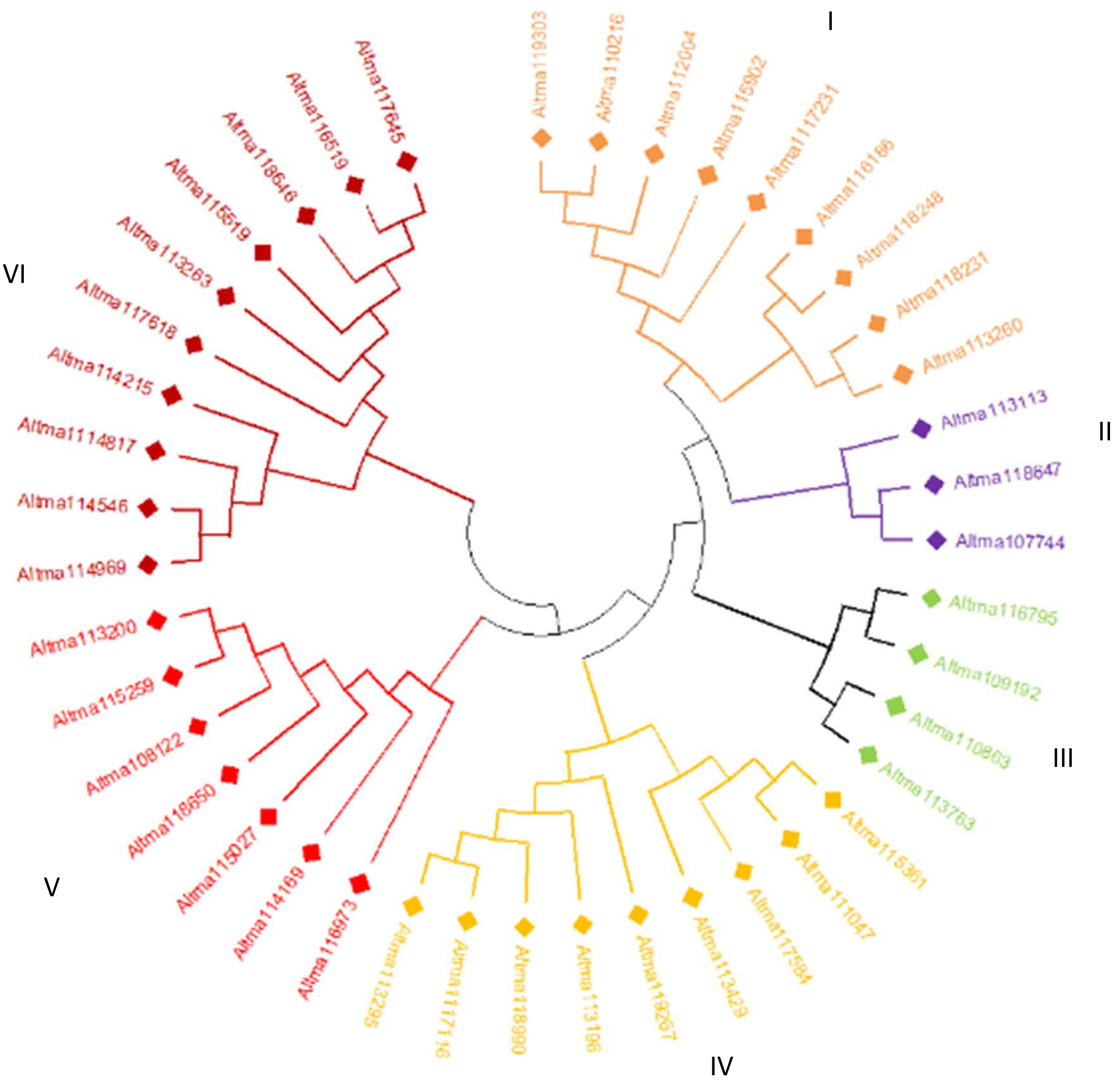

Alternaria macrospora

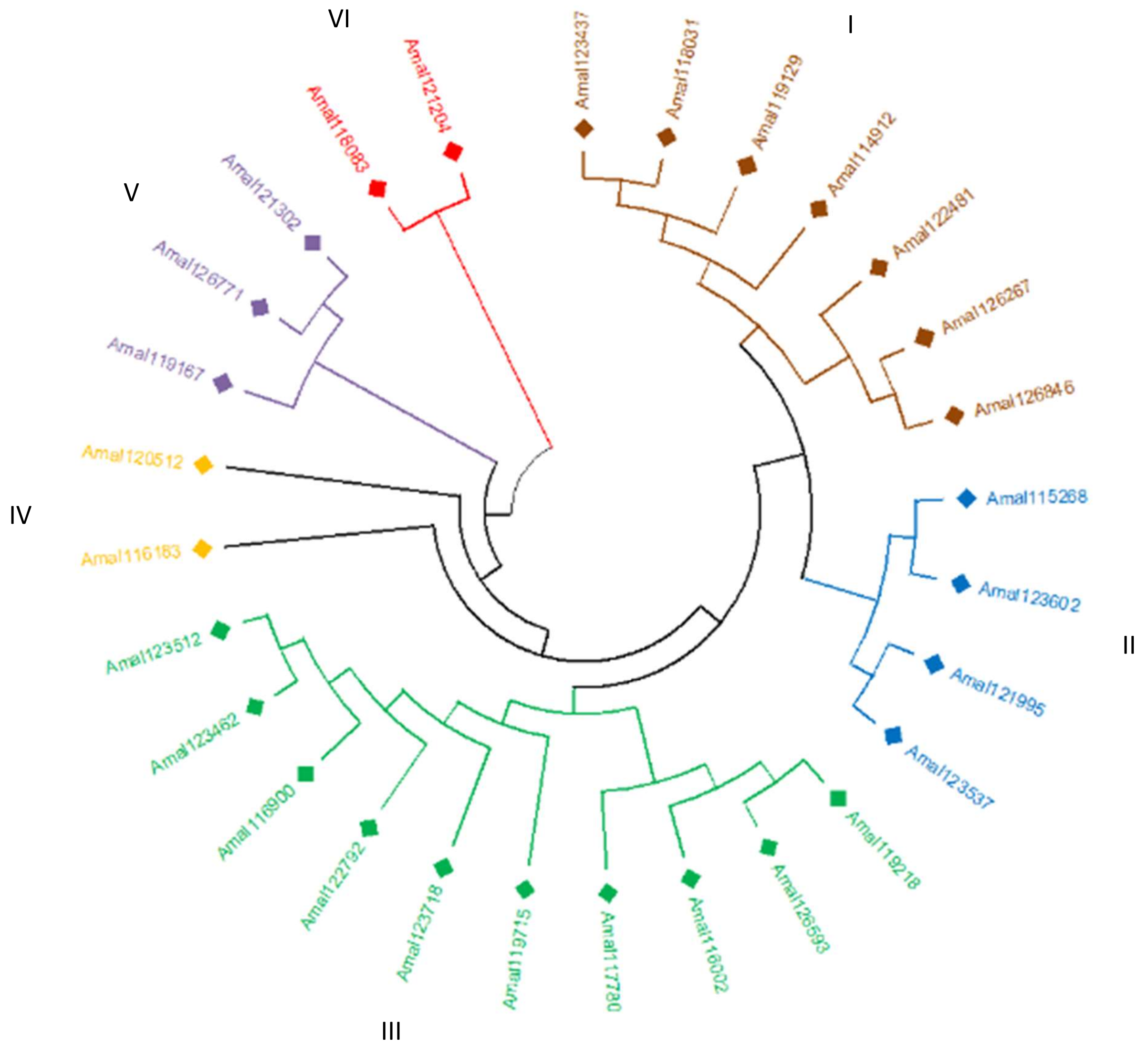

Alternaria mali

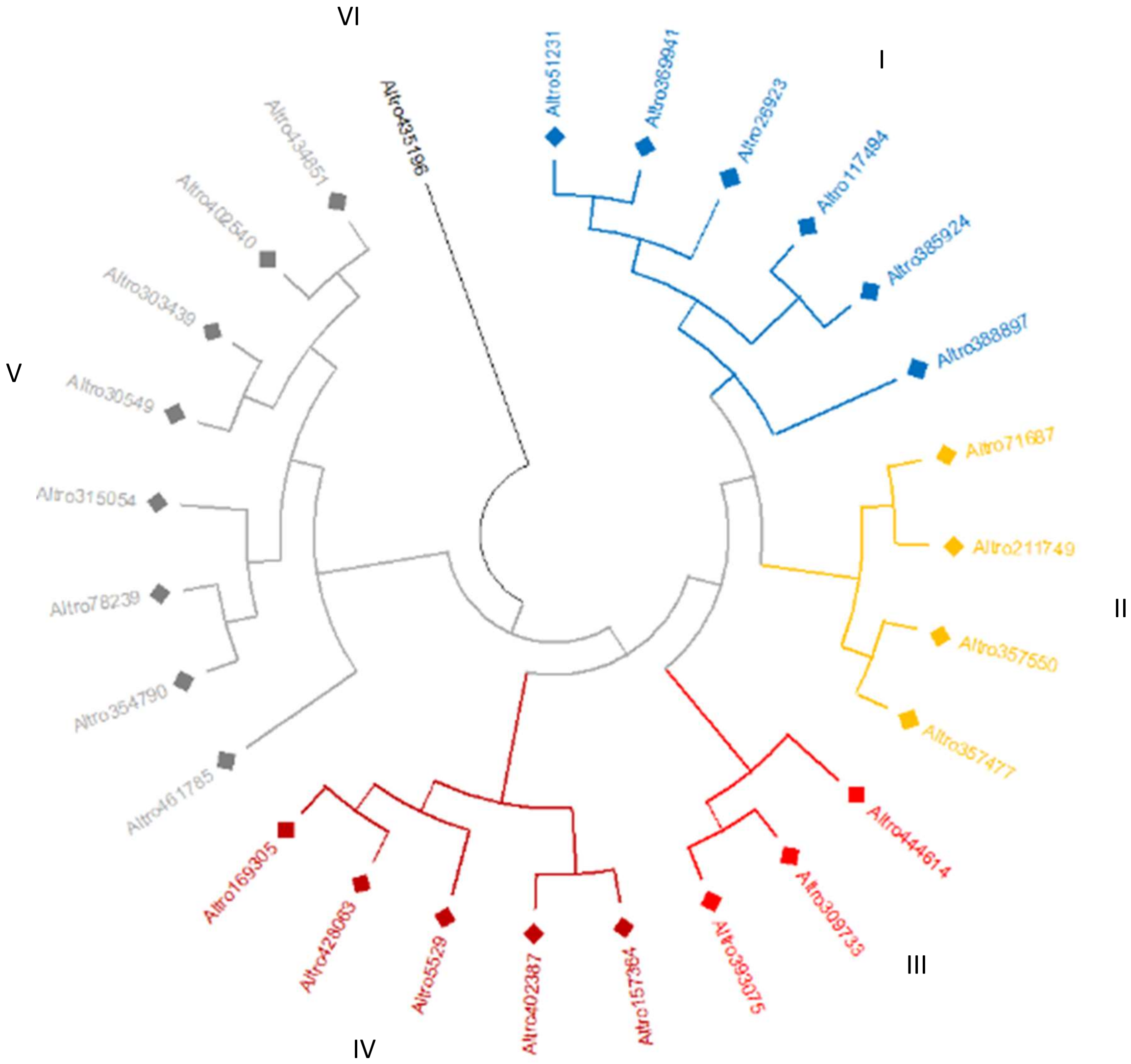

Alternaria rosae

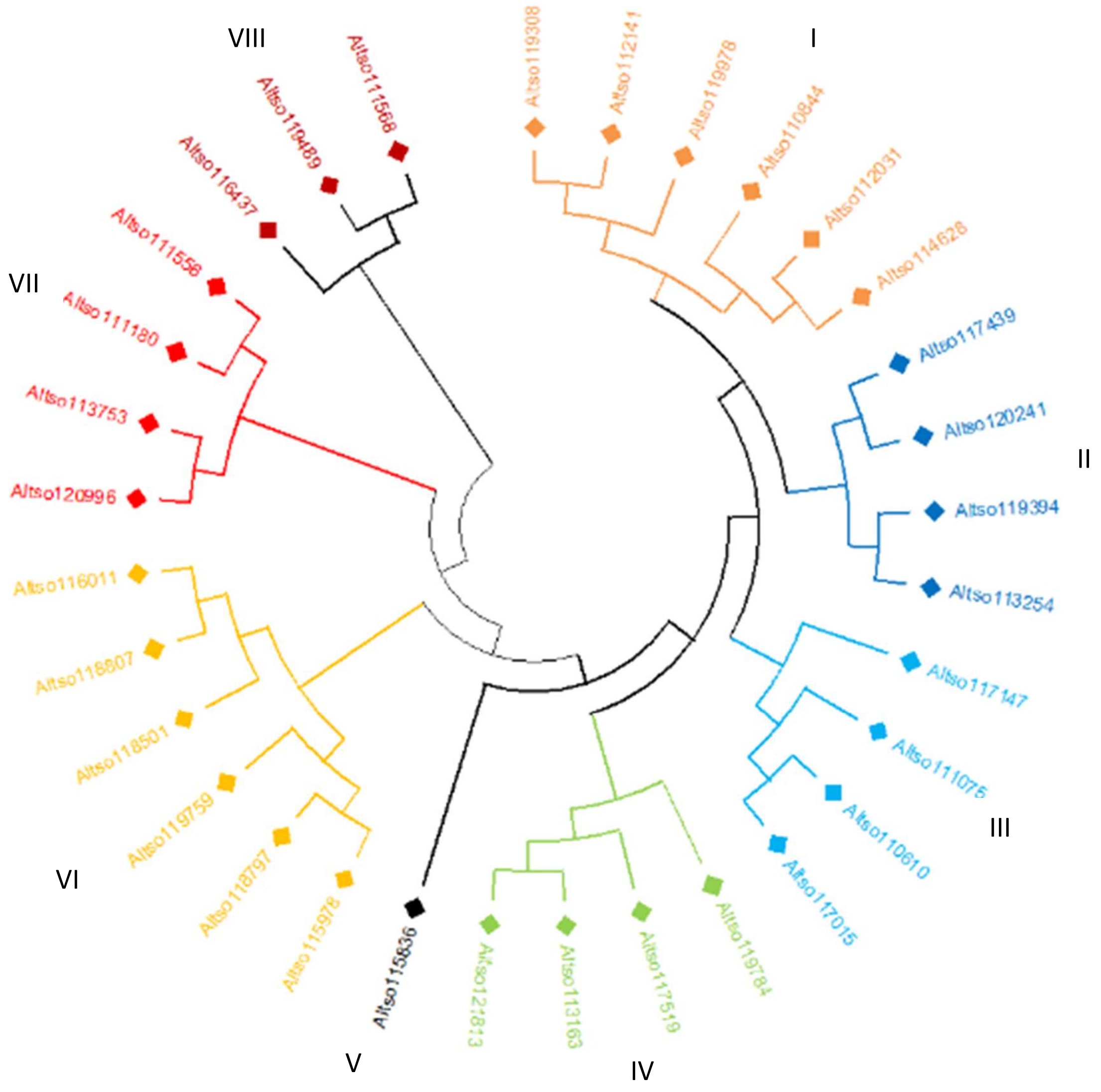

Alternaria solani

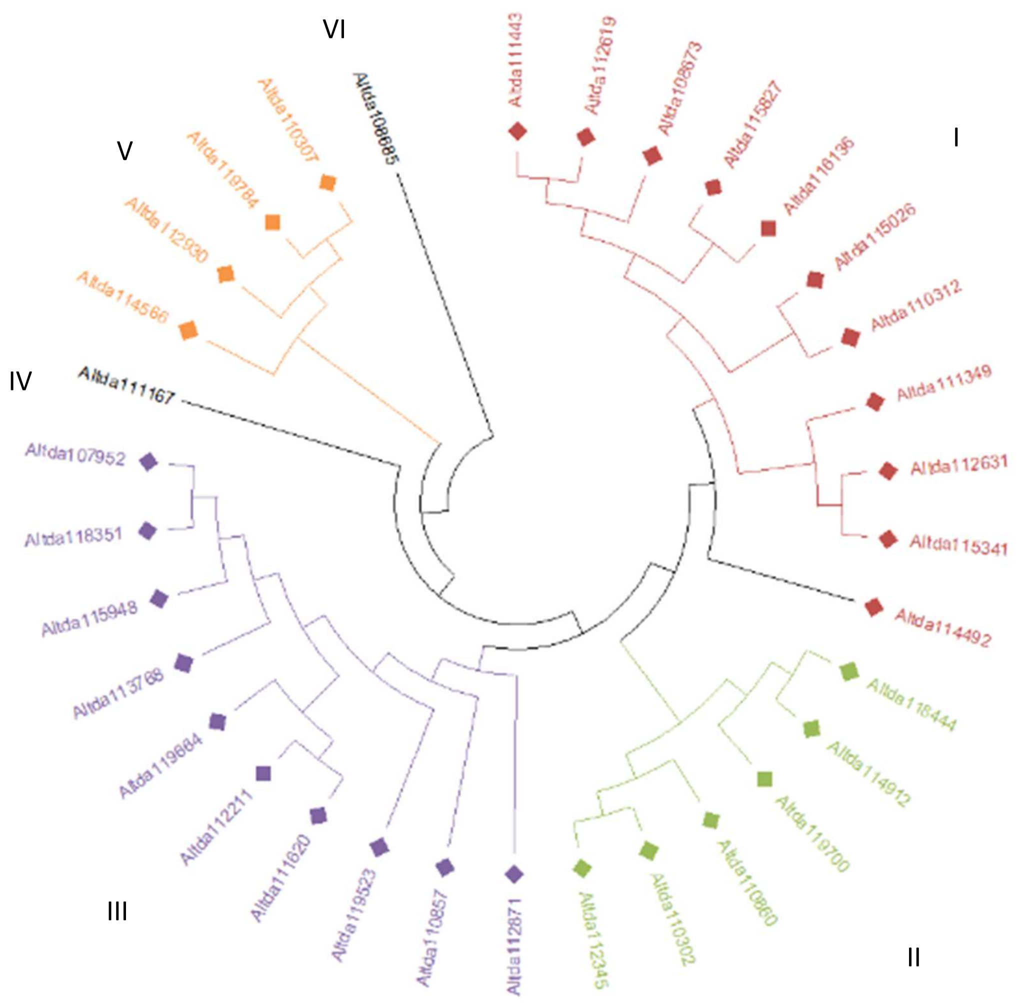

Alternaria dauci

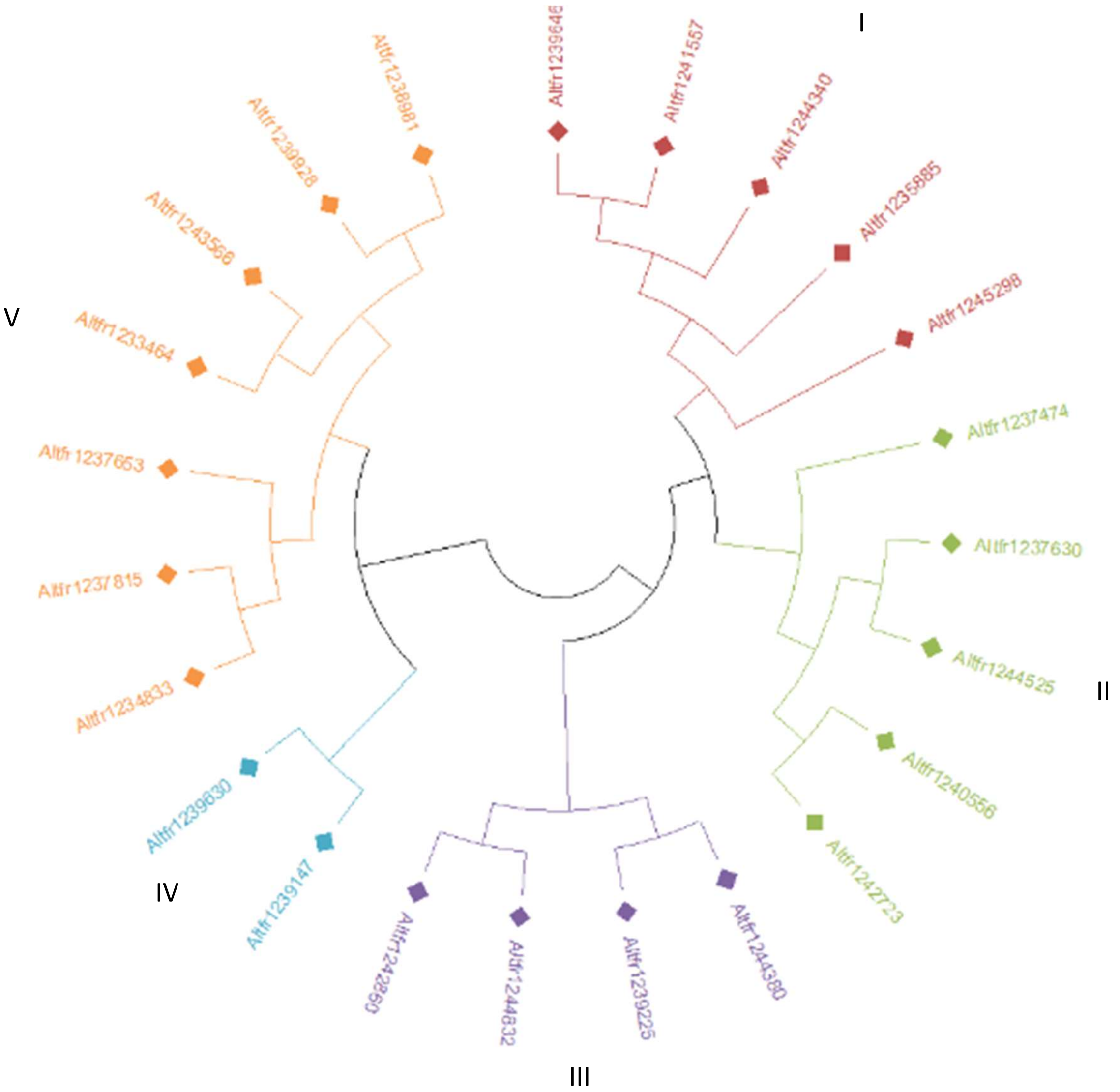

Alternaria fragaria

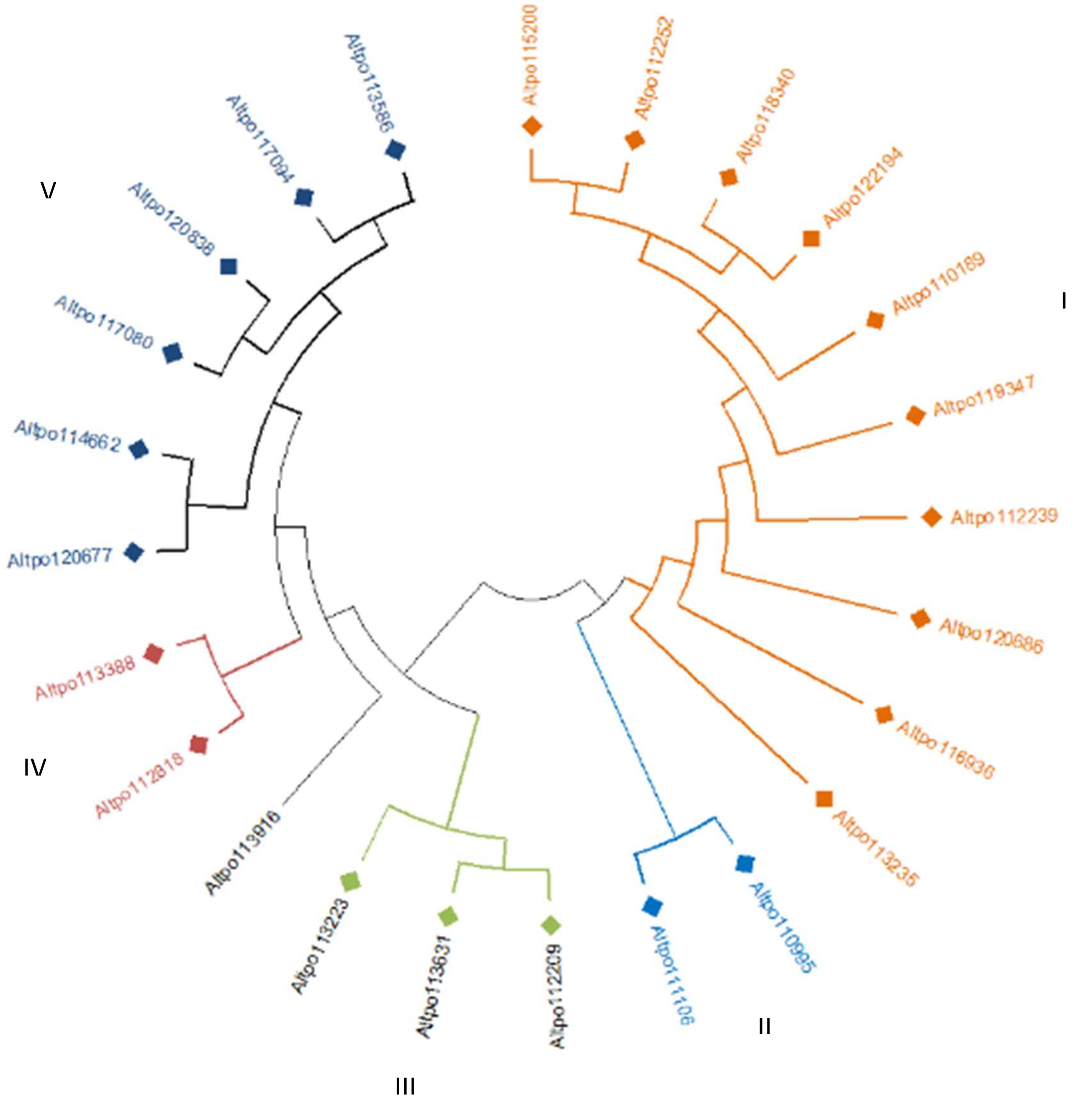

Alternaria porri

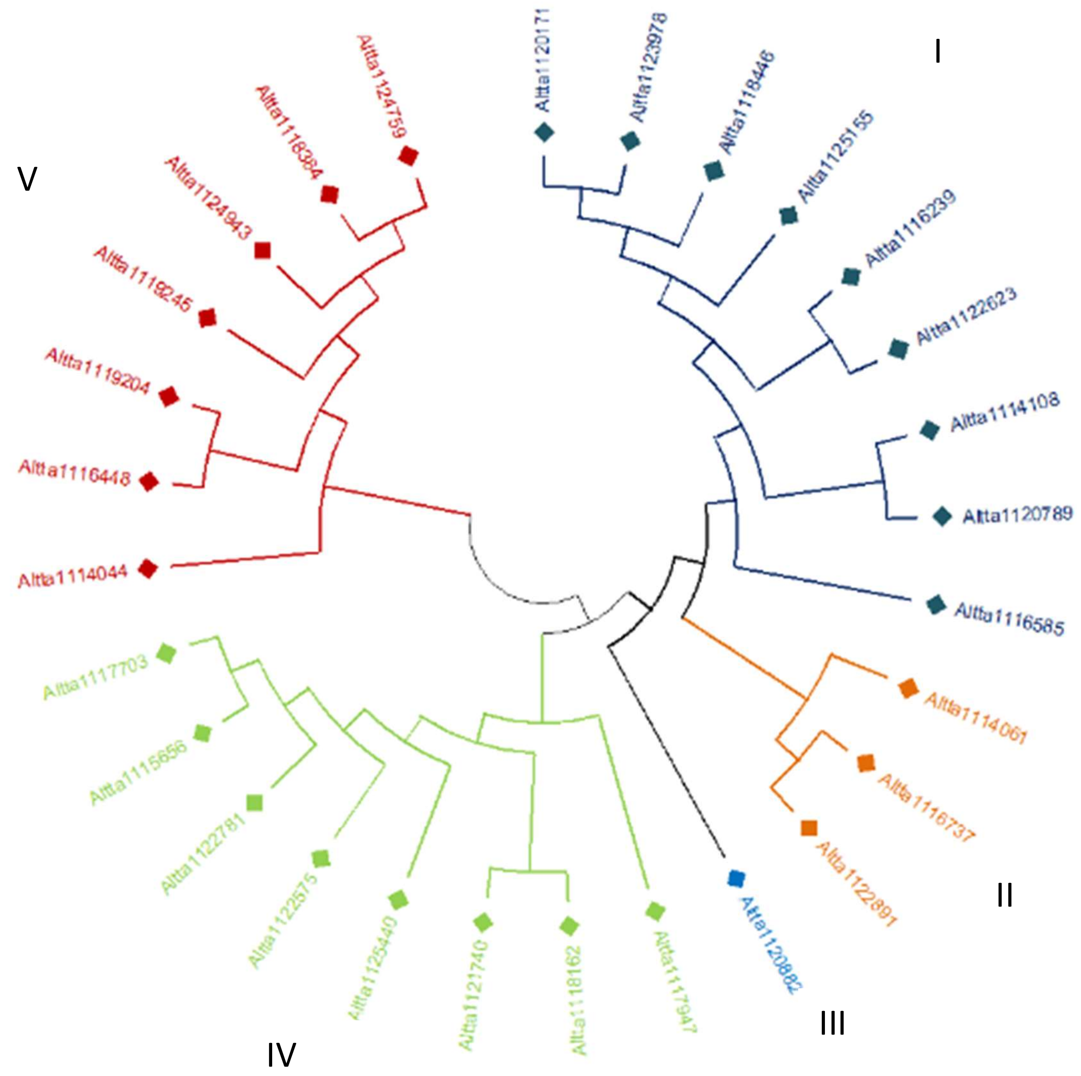

Alternaria tangelonis
